# Inkjet-Printed, Flexible Organic Electrochemical Transistors for High-Performance Electrocorticography Recordings

**DOI:** 10.1021/acsami.4c07359

**Published:** 2024-08-15

**Authors:** Fadi Khoury, Sahera Saleh, Heba Badawe, Makram Obeid, Massoud Khraiche

**Affiliations:** † Neural Engineering and NanoBiosensors Group, Biomedical Engineering Program, Maroun Semaan Faculty of Engineering and Architecture, American University of Beirut, Beirut 1107 2020, Lebanon; ‡ Stark Neurosciences Research Institute, Department of Neurology, Indiana University School of Medicine, Indianapolis, Indiana 46202, United States

**Keywords:** amplification, electrocorticography, neural interface, Organic Electrochemical Transistor (OECT), seizure

## Abstract

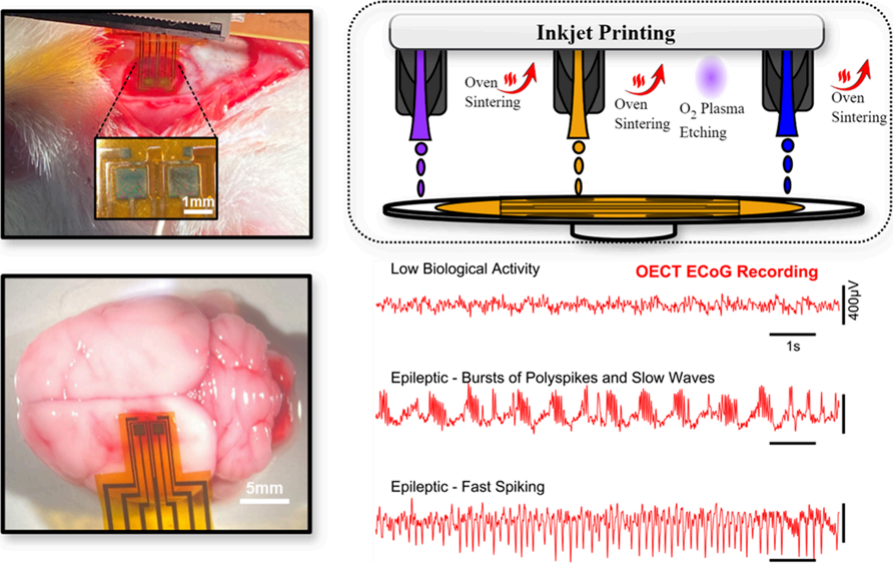

Organic electrochemical transistors (OECTs) have emerged as powerful tools for biosignal amplification, including electrocorticography (ECoG). However, their widespread application has been limited by the complexities associated with existing fabrication techniques, restricting accessibility and scalability. Here, we introduce a novel all-planar, all-printed high-performance OECT device that significantly enhances the accuracy and sensitivity of ECoG recordings. Achieved through an innovative three-step drop-on-demand inkjet printing process on flexible substrates, our device offers a rapid response time of 0.5 ms, a compact channel area of 1950 μm^2^, and is characterized by a transconductance of 11 mS. This process not only simplifies integration but also reduces costs. Our optimized in-plane gate voltage control facilitates operation at peak transconductance, which elevates the signal-to-noise ratio (SNR) by up to 133%. In vivo evaluations in a rat model of seizure demonstrate the device’s performance in recording distinct electrographic phases, surpassing the capabilities of PEDOT:PSS-coated gold-based ultralow impedance passive electrodes, achieving a high SNR of 48 db. Our results underscore the potential of Inkjet-printed OECTs in advancing the accessibility and accuracy of diagnostic tools that could enhance patient care by facilitating timely detection of neurological conditions.

## Introduction

Since their seminal introduction by White et al. in 1984, Organic Electrochemical Transistors (OECTs) have become essential components in cutting-edge applications, particularly in biointerfacing electronics.^1^ OECTs function through dual voltage controls (Figure S1): the drain-source voltage (V_DS_) drives current within the channel, while the gate-source voltage (V_GS_) modulates the semiconductor’s conductivity via an aqueous electrolyte.^2,3^ At their core, Organic Mixed Ionic Electronic Conductors (OMIECs), like poly(3,4-ethylenedioxythiophene) doped with poly(styrenesulfonate) (PEDOT:PSS), endow OECTs with the ability to interface with biological processes, offering high stability and resolution especially with high carrier mobility and volumetric capacitance.^4^ This composition makes the channel’s capacitance dependent on its thickness rather than the typical cross-sectional area, yielding high transconductances (g_m_) at optimal gate voltages (V_GS,gm(max)_). These transconductances significantly surpass those of organic field-effect transistors (OFETs).^3^ Biocompatible and resilient across varying temperatures and pH levels, OECTs are particularly advantageous for low-voltage (<1 V) operations and show inherent ion sensitivity.^5−7^ These characteristics make them highly effective for biointerfacing applications, from molecular sensing to recording electrophysiological activities in neural and cardiac contexts.^8−15^ Of critical importance, OECTs excel in amplifying brain signals for Electrocorticography (ECoG), an area where high-fidelity signal tracing is essential for accurately localizing the sources of pharmacoresistant seizures amenable to therapeutic and resection interventions.^16^ Careful selection of flexible substrates on which devices are fabricated ensures intimate contact with the surface of the brain. However, the conventional fabrication of high-performance OECTs often involves complex and costly cleanroom microfabrication processes. These traditional methods not only generate substantial waste but also involve the use of harsh chemicals and high-temperature steps, posing risks to users and to heat- and chemically sensitive materials like ultraflexible, low-melting temperature polymers.^17^ Additionally, achieving precise device configurations, such as selectively coating the gate terminal in a planar layout, adds to the complexity.^18^

In this study, we develop an innovative three-step fabrication approach to build a high-performance, flexible, and stable OECT device designed for electrocorticography recordings, utilizing contactless Drop-on-Demand (DoD) Inkjet printing. Here, we present a detailed analysis of the electrical performance of our device, benchmarking it against existing technologies, and demonstrate its superior capability in detecting hippocampal seizure signatures in vivo, induced by the chemoconvulsant kainic acid (KA). Our approach not only simplifies the fabrication process but also significantly reduces the cost and environmental impact associated with traditional methods.

## Experimental Section

### Materials

Gold nanoparticle ink (JG-125 Novacentrix, USA) was used to pattern the OECT terminals and the electrodes. Inkjet printable Polyimide ink was used to insulate (PI-IJ, UT DOTS, USA). Inkjet printable PEDOT:PSS was used as the semiconducting channel (Sigma-Aldrich, USA). Phosphate Buffer Saline (PBS) was purchased from Sigma (PBS, P4417). All inks were used as is without modification. One Mil Kapton polyimide films were used as the printing substrate (CS Hyde, USA).

### Fabrication

**Scheme 1:**
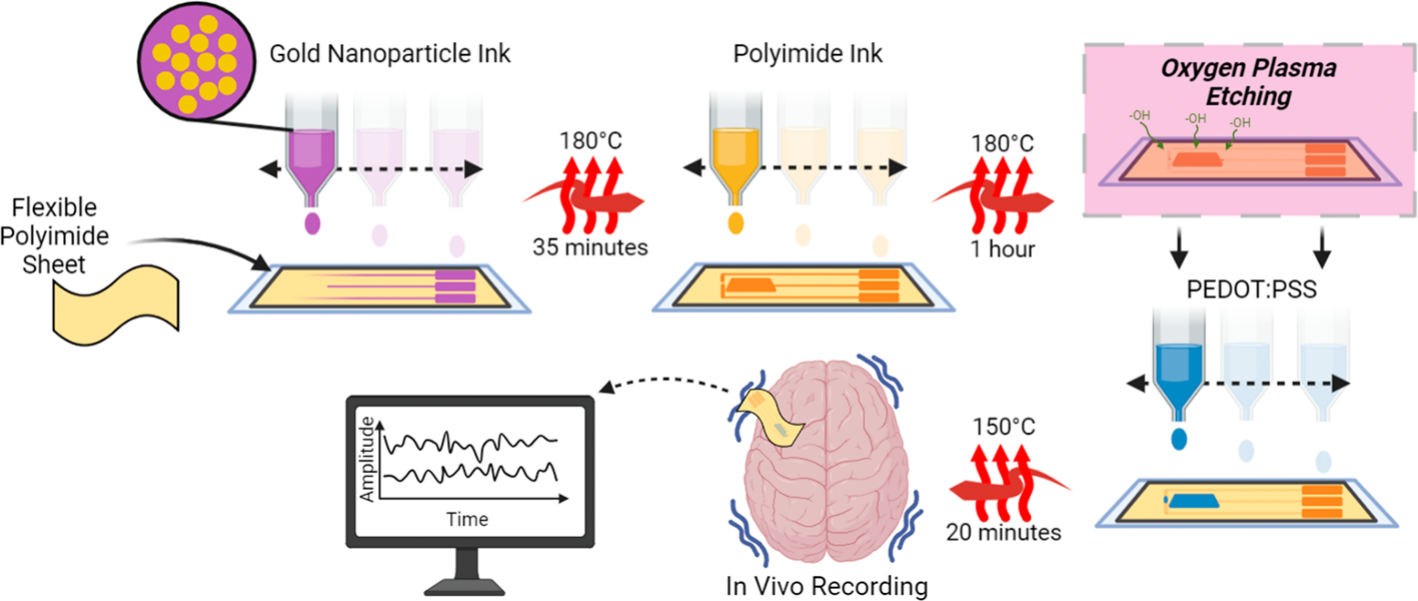
fabrication flow of inkjet printed OECTs. Gold is first deposited to pattern the conductive leads. Polyimide is then used to insulate. The insulated device is then treated with oxygen plasma etching to increase surface wetting. Finally, PEDOT:PSS is deposited

The Dimatix Materials Printer (DMP-2850, Fujifilm, Dimatix, USA) fitted with a 2.4 pL piezo-driven 12-nozzle printhead (Samba, PDS00142) was used to fabricate devices on flexible polyimide sheets. Detailed explanation of the ink preparation, Drop Spacing (DS), jetting waveform, and postprinting processing is explained in previous work^19^ and in Figure S2. Briefly, the fabrication consists of three main steps following substrate cleaning and dehydration. First, metal patterning by depositing the gold ink (DS 20 μm, sintering at 180 °C for 35 min), Then insulation is achieved by covering the leads with polyimide ink (DS 15 μm, curing at 180 °C for 1 h), and finally the semiconducting channel is formed by layering PEDOT:PSS (DS 15 μm, curing at 150 °C for 20 min). Prior to depositing the semiconductor, oxygen plasma etching (vacuum set point: 499.3 mTorr, RF power: 150 W, oxygen flow rate: 15 cc/min) is performed for a duration of three seconds to achieve better film uniformity and to avoid gaps in the prints, this is achieved using the PE-25 model from PlasmaEtch. A sketch depicting the fabrication flow of an Inkjet OECT neural interface is shown in Scheme 1.

### Device characterization

#### Electrical

National Instrument (NI) Source Measure Units (SMU, PXIe-4138) were used to scan for I–V curves, transfer characteristics, and TLM measurements. A waveform generator (PXIe-5413) was used for tests requiring AC modulations. Control of the SMU units was performed using custom LABVIEW Virtual Instrument (VI) code files. All electrical characterizations in solution were conducted in 1X PBS using either an Ag/AgCl external probe or a printed planar gold/gold-coated electrode as the gate electrode.

#### Electrochemical Impedance and Cyclic Voltammetry Analysis

The Electrochemical performance of the bare gold and PEDOT:PSS coated gold electrodes was evaluated using the Interface 1010T from Gamry Instruments. All measurements were conducted in a three-electrode cell with 1X PBS, using a platinum electrode as counter electrode (CHI102, CH Instruments, United States) and Ag/AgCl as reference electrode. Electrochemical Impedance Spectroscopy (EIS) was measured with an AC voltage of 50 mV rms with frequencies ranging from 1 Hz to 20 kHz at 10 points/decade. Cyclic voltammetry is performed at a scan rate of 1000 mV/s with a scan limit from −0.9 to +0.9 V, a step size of 2 mV, and a cycle number of ten.

#### Dimensions

Device photographs were captured using the fiducial camera of the inkjet printer, and an optical microscope (8X). A Digital holographic microscope (DHM, LyncéeTec, Switzerland) was used to measure the 3D dimensions of the developed thin films and extract the cross-sectional dimensions along the 2D axes.

#### Contact Angle

A goniometer OCA 15EC (Dataphysics, Germany) was used to examine the morphological interaction of PEDOT:PSS with pristine and modified (oxygen plasma etched) PI films by assessing the contact angle of with the sessile drop method. The SCA20 software (Dataphysics, Germany) was used to measure angles. Wettability was measured by dripping 5 μL of PEDOT:PSS droplets to each surface at a speed of 1 μL sec–1 and measuring the angle formed between the droplet and the surface of the sample. Measurements were taken 10 s after the droplets were introduced to maintain consistency. Four different PI samples were analyzed per configuration with four drops each.

#### Electrical Characteristics

Device characterization data was collected using LABVIEW. The transmission line model (TLM) technique was used to assess the contact resistance between gold or silver with PEDOT:PSS by applying a 1 mV DC voltage between 3 × 6 mm rectangular metal pads connected by the semiconductor. The response time was extracted from the device’s current response to a step voltage of 20 Hz with limits from −0.5 V_GS_ to 1 V_GS_, ensuring maximum and minimal channel conductivity, respectively. When evaluating the difference in OECTs and electrodes when recording a 1 Hz sine wave, Signal-to-Noise Ratio (SNR) was calculated by taking the difference between the decibel power of the one-Hz frequency and the average power of the rest of the FFT spectrum. To feed the OECT neuronal spikes, a signal generator (60MEA2100-SG) purchased from Multichannel Systems was used, with the signal source setting set at hippocampal slice population spikes. Data collection in vivo was performed using the ME2100-system purchased from Multichannel Systems with the integrated analog band-pass filter set from 1-to-500 Hz. The data was treated offline with a 200 Hz digital lowpass filter and a 50 Hz notch filter. To generate scalograms, the Morlet wavelet was used. Analysis and plotting of all data were done using a custom MATLAB R2023b code.

### Animal Model

All experiments were approved by the Institutional Animal Care and Use Committee (IACUC) at the American University of Beirut. Adult male Sprague–Dawley rats, weighing 300–350 g on average (n = 3) were used for electrocorticographic recordings. After anesthesia via intraperitoneal injection of Ketamine (80 mg/kg) and Xylazine (20 mg/kg), the rat’s head is shaved from the region between the eyes down to its ears. The animal is then carefully mounted on a stereotaxic apparatus by placing ear bars in both auditory meatuses and securing the animal’s head with a mouthpiece set at the appropriate height for adult rats. Afterward, the animal’s head is scrubbed with isopropyl alcohol and betadine. Then, using a scalpel, an incision is made down the midline and clamps are used to hold the skin away from the skull. A sterile swab is then used to remove underlying tissues adhering to the skull to expose the bregma and lambda. After identifying the coordinates of bregma, the four edges of the craniotomy site are identified, where the area is set to be approximately 4 × 4 mm on the left side of the brain above the somatosensory area. A surgical drill is then used to drill off the bone surface, after which the dura is removed using fine-tip forceps. For seizure induction, intra-amygdalar KA (Abcam, Cambridge, MA, USA) injection was done prior to placement of the interface on the cortex. 0.6 μL of 1 mg/mL (KA in saline) is injected into the amygdala (−2.8 mm Anteroposterior (AP), 5 mm Mediolateral (ML), and 8.8 mm Dorsoventral (DV, from the skull)) using a 1 μL Hamilton syringe fixed to a stereotaxic holder.^20^ The interface is then carefully placed on the surface of the cortex and connected to the data acquisition system. Recordings are then obtained for a period of 1 h after KA injection.

## Results and Discussion

### Fabrication and Dimension

For optimal OECT performance, the channel dimensions were chosen with careful consideration of the limitations imposed by the application, these dimensions play a vital role in the device’s success in detecting and measuring biological signals. For electrophysiological ECoG applications, the device must have high spatial resolution (900 um or less is often desired to resolve individual cortical columns), high temporal resolution (microseconds to milliseconds), high amplification, and a wide operational bandwidth (0.1 to 500 Hz).

Utilizing Inkjet microfabrication, the device’s dimensions can be controlled via adjusting the programmed patterns according to the equation:
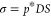
1With σ representing either the x or y 2D dimension, *p* is the design’s pixel number, and *DS* is the set drop spacing. To estimate process’s accuracy in lead widths, five, 1 layer, 100 μm lines were printed and scanned with the DHM. The average printed line width was 104.5 μm with a standard deviation of 0.8 μm. This corresponds to an absolute error of 4.5 μm and a relative error of 4.5%, indicating an accuracy of 95.5%. The precision, indicated by the standard deviation and the accuracy, shows good consistency in the printed line widths. In addition, the accuracy of lead separation that is relevant to the channel length is also tested, for a design of 20 μm the average length was 19.5 μm with a standard deviation of 0.7 μm, resulting in an accuracy of 97.5% (Figure S3).

The physical attributes of the device affect g_m_ of a depletion mode OECT in the saturation regime following:
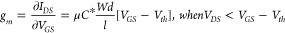
2Where μ is the hole mobility, C* is the channel’s volumetric capacitance, *W* is channel width, *d* is channel thickness, *l* is channel length, and *V*_*th*_ is the device’s threshold voltage.^2^ Furthermore, the device’s response time is determined by
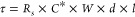
3Where τ is the response time and *R*_*S*_ is the resistance of the electrolyte. Equations 2 and 3 reveal a trade-off between gain and speed, requiring a careful balance tailored to the specific application.

Reducing the channel length significantly improves device performance in terms of response time and transconductance (Eqs. 2 and 3). This enhancement is governed by two primary factors in inkjet printing: the distance between adjacent metal ink droplets (drop spacing, DS), and ink spreading. We minimized the source-drain gap of the device to a single pixel. The optimal drop spacing of the gold ink, set at 20 μm, determines the smallest achievable channel length, which matches the drop spacing (Eq. 1).

**Figure 1:**
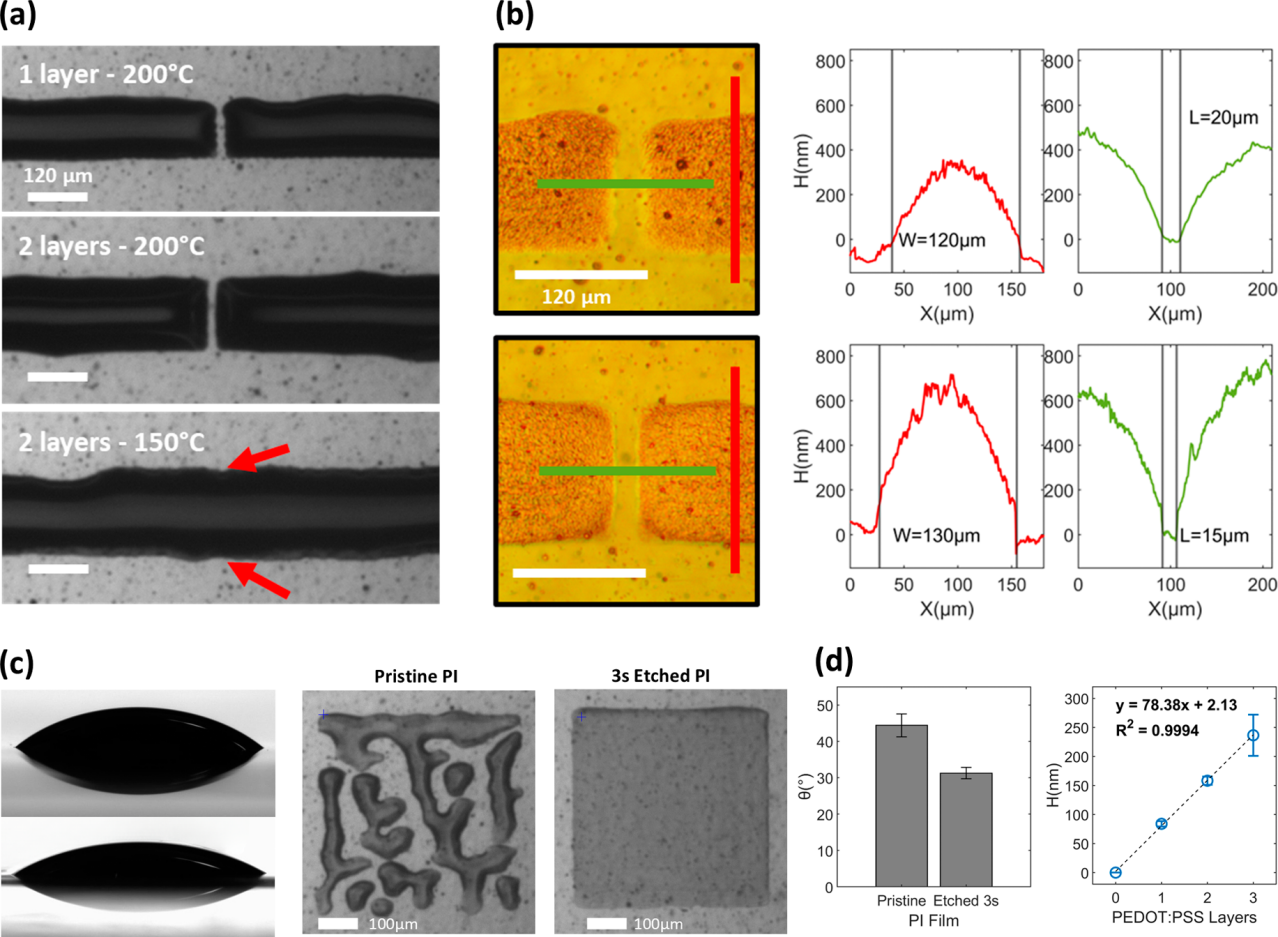
(a) Effect of preheating the substrate prior to metal deposition at 200 and 150 °C and its effect on the source drain separation, red arrows indicate the intended position of the source and drain separation. (b) Microscopic view of the source and drain metals with DHM thickness profiling lines (length in green, width in red). (c) Sessile drop of PEDOT:PSS with pristine and etched PI and the corresponding effect on print uniformity. (d) Contact angle of PEDOT:PSS with polyimide (left) and the thickness of inkjet-printed PEDOT:PSS function of the number of layers (right).

Preparation of the substrate before ink deposition is critical for ensuring high print resolution. Our study utilizes water-based metal inks, underscoring the importance of preheating the substrate just before fabrication. This step, conducted well above the boiling point of water, dehydrates the substrate, facilitating controlled ink deposition and preventing excessive spreading. Figure 1a illustrates the adverse effects of overspreading when the temperature is too low. Treating the polyimide at 200 °C for 30 min proved to be the most efficient approach.

While increasing the platen temperature is an option in inkjet fabrication, it can counteract substrate dehydration when using water-based inks due to the inverse relationship between water contact angle and temperature.^21^ Nonetheless, controlled ink spreading can be advantageous, particularly for regulating the length of the OECT channel beyond the one-pixel limit. By depositing a secondary metal layer, ink spreading resulted in highly consistent channel lengths of 15 μm across multiple fabrication iterations.

Channel width is also a critical determinant of performance. Excessive width limits device speed and pushes V_GS,gm(max)_ to more positive values, while insufficient width restricts transconductance. We determined an optimal channel width of 120 μm, which effectively increased to 130 μm after spreading due to the second layer (Figure 1b).

Regarding thickness, engineering OECTs with a thin channel enhances switching speeds and bandwidth. However, this may reduce transconductance and increase susceptibility to nonuniform film distribution. Depositing PEDOT:PSS on pristine flexible PI films initially resulted in nonuniform prints due to droplet adherence or clustering. To achieve uniform film deposition, we modified the substrate with a 3-s oxygen plasma etching process to increase PI wettability (Figure 1c). This process decreased the contact angle between PEDOT:PSS and PI from 44 ± 3° to 31 ± 1.5°. Consequently, layering PEDOT:PSS led to a 99.94% linear increase in thickness of approximately 78 nm per layer (Figure 1d).

### Electrical

#### In Plane Gate and Efficient Gating

In electrophysiological monitoring, such as ECoG, the signal of interest is often small in magnitude and easily obscured by noise. To enhance the SNR of recorded signals, optimal OECT performance is crucial, achieved by setting the gate voltage to V_GS,gm(max)_. While a three-dimensional off-device integration of the gate terminal provides control over gate voltage, it introduces complexities due to concerns about biofouling and scarring in an in vivo setting.^22^ Alternatively, a planar integration of a biocompatible inkjet printed electrode would ensure peak g_m_ operation, leading to better sensitivity in detecting signals while simplifying the fabrication and in vivo implantation of the interface.

**Figure 2:**
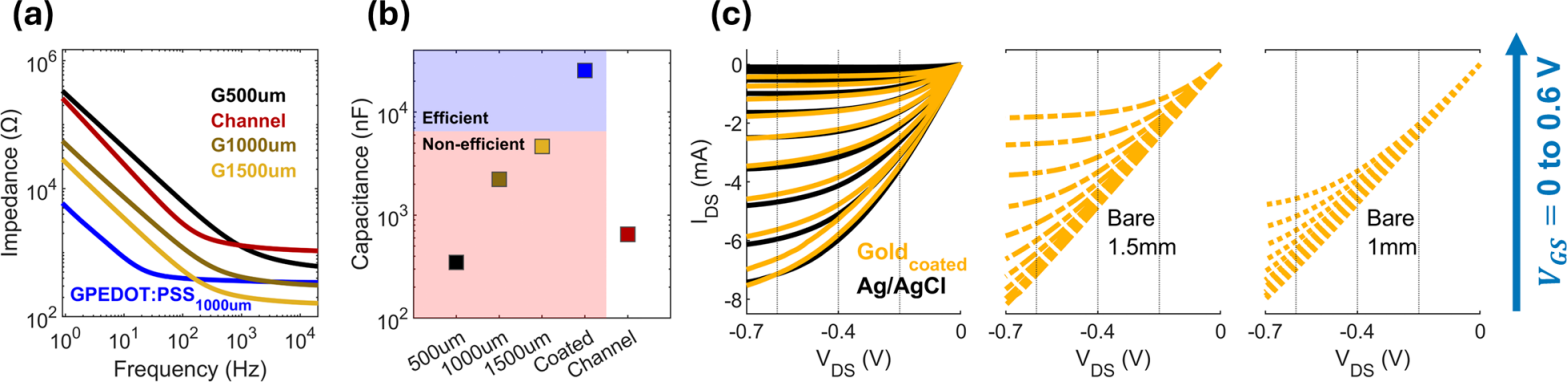
(a) Electrochemical impedance spectroscopy of an OECT channel and four gate configurations, including three bare square electrodes of sizes 500, 1000, and 1500 μm and one PEDOT:PSS coated 1000 μm gold electrode. (b) Capacitances extracted from the simplified randles model. (c) IV curves of OECTs, gated with a 1 mm (right), 1.5 mm (middle), gold coated (left, in yellow), and Ag/AgCl (left, in black) electrode.

However, selecting the appropriate material for the gate presents a challenge, especially considering the polarizable nature of materials like a biocompatible printed gold electrode. This raises concerns about the voltage drop across the gate/electrolyte interface, potentially resulting in inefficient OECT gating and consequently reducing sensitivity, represented in Figure S1b.^3,23−25^ Addressing these challenges requires modifications to the gate structure to achieve both efficiency and compactness. To efficiently gate the device, an electrode with similar characteristics to that of a nonpolarizable (Ag/AgCl) electrode having zero voltage drop must be developed.^26^ Rivnay et al. reported that a gate to channel capacitance ratio above 10 is sufficient for efficient gating.^2^ This is further modeled by Bernard, et al, where increasing the capacitance at the gate would make the voltage drop negligeable to the one observed at the channel and can therefore be ignored in the proposed ionic circuit, allowing us to assume no voltage drop at the gate/electrolyte interface.^27^ Increasing gate electrode size is one approach to boost capacitance, yet it reduces spatial resolution. Alternatively, modifying the gate by coating it with a polymer boasting high volumetric capacitance offers a solution. This method can achieve high capacitance while conserving square footage. The Inkjet Printing method allows for precise control over electrode size and coating thickness, with the latter capable of easily reaching heights of up to several micrometers, surpassing what is achievable via conventional microfabrication techniques. To demonstrate the effect of electrode size and polymer coating thickness on capacitance, and consequently, efficiency in gating OECTs, we evaluated the performance of four-square shaped inkjet-printed electrodes comprising three bare gold electrodes sized at 500 μm, 1000 μm, and 1500 μm, alongside a single gold electrode (1000 μm) coated with PEDOT:PSS (900 nm) placed 100 μm away from the channel. To extract the capacitance of each structure, impedance spectroscopy was used, and the curves were fitted using a simplified Randles model:
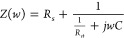
4Where *R*_*s*_ is the resistance of the solution, *R*_*ct*_ is the charge transfer resistance and *C* is the capacitance. Figure 2a shows that increasing the size of the gate from 500 to 1500 μm leads to a notable reduction in impedance. For instance, at 1 kHz, the impedance values decrease, measuring 1.2 kΩ, 1.4 kΩ, and 0.2 kΩ for gate sizes of 500 μm, 1000 μm, and 1500 μm, respectively. Also, our results show that the coated configuration is more efficient at decreasing impedance than increasing the electrode size, especially at low frequencies were charge transfer resistance dominates the behavior of the system.^28^ All bare gold gate sizes fail to satisfy the capacitance ratio needed for efficient gating of the OECT channel. On the contrary, the coated configuration surpassed the desired threshold (Figure 2b).

**Figure 3:**
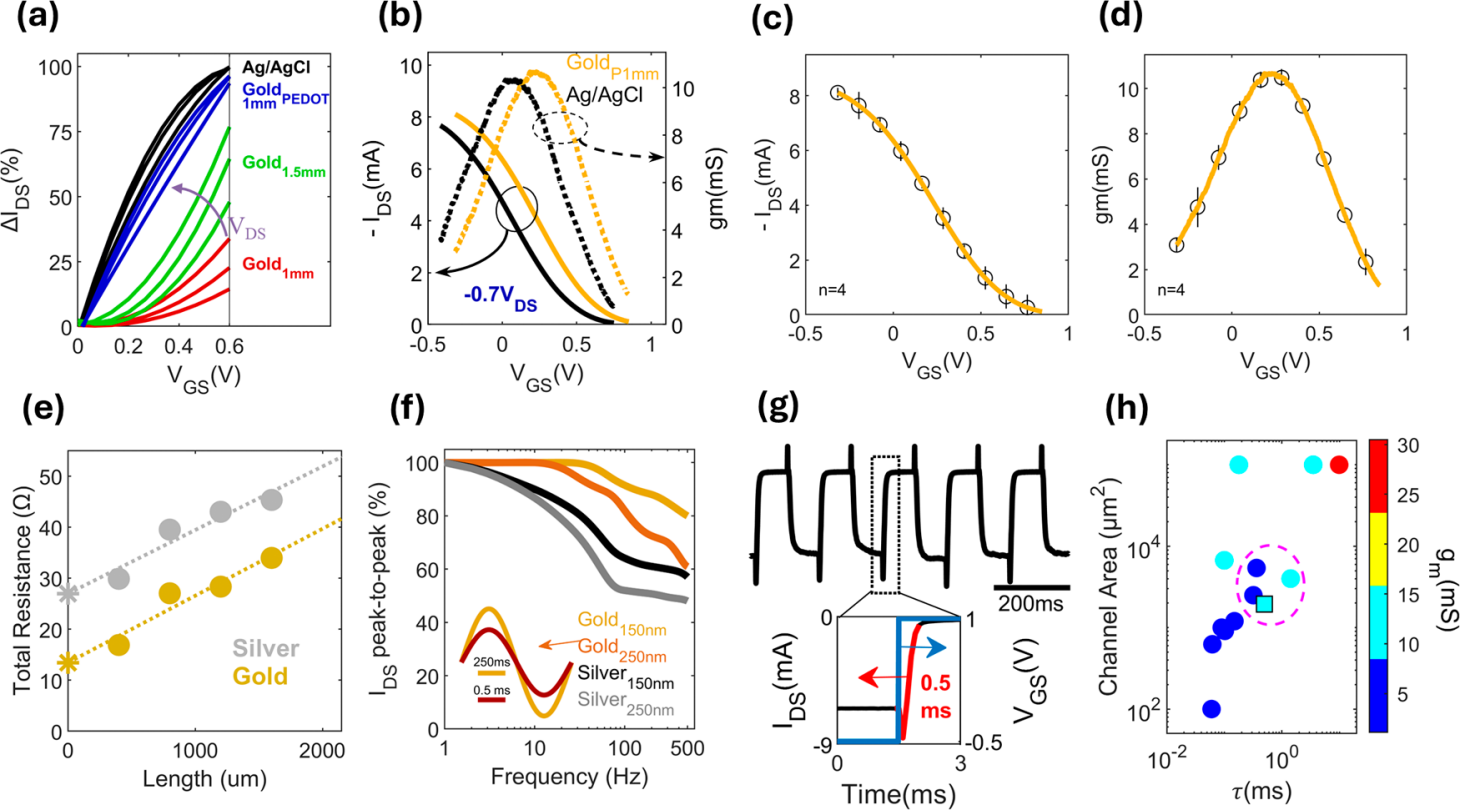
(a) I_DS_ modulation at 0.2Vds, 0.4Vds, and 0.6Vds from the IV curves demonstrating efficient gating from the PEDOT:PSS coated gold gate. (b) Transfer characteristic of the OECT device using an Ag/AgCl electrode or a PEDOT:PSS coated gold electrode. (c) Averaged transfer characteristic and (d) g_m_ curves of four inkjet printed OECTs on polyimide. (e) TLM measurements to evaluate the contact resistance of gold and silver with PEDOT:PSS, round scatter dots represent measured data, dotted lines are the best fit lines, and the star symbol is the interaction of the best fit line with the *y*-axis, corresponding to the contact resistance. (f) Frequency dependent amplitude drop of a device using gold and silver source and drain at channel thicknesses of 150 and 250 nm. Inset figure is a representation of the amplitude drop between 1 and 500 hz for a gold source and drain setup at 250 nm. (g) Current step response of the OECT, inset figure highlights the applied step voltage, the response time of 0.5 ms, and the on and off currents. (h) Performance comparison of the fabricated device against high performing OECTs in literature considering channel area, response time, and transconductance. dashed pink circle represents the devices used for monitoring brain activity, square data point represents our work.

Analysis of the PEDOT:PSS based OECT I–V curves show typical depletion mode transistor behavior (Figure 2c), and extraction of currents at −0.2 V_DS_, −0.4 V_DS_, and −0.6 V_DS_ reveal efficient modulation of channel conductivity by the coated configuration only, akin to gating behavior with an Ag/AgCl electrode (Figure 3a). A precise and efficient control over channel conductivity is particularly significant when utilizing transistors as amplifiers since inefficient gating not only diminishes peak g_m_ but also shifts it to more positive gate biases. This compromise in performance increases power demand and noise levels, ultimately leading to signal distortion and degradation of signal fidelity. Notably, the corresponding transfer characteristic obtained with the coated gold gate exhibits a positive shift of V_GS,gm(max)_ with an insignificant change in peak g_m_ compared to the Ag/AgCl setup and reaching a peak of 11mS (Figure 3b). To ensure correct analysis of the transfer characteristic and consequently correct extraction of the peak g_m_, the set gate voltage sweeping rate of 0.06 V/s is confirmed to lead to a minimal hysteresis when sweeping in the forward and backward direction (Figure S4). Across four fabrication iterations, the inkjet printed devices showcase highly reproducible performance metrics, underscored by the minimal standard deviation error bars when examining transfer characteristics (Figure 3c) and g_m_ curves (Figure 3d).

#### Bandwidth and Response Time

Bandwidth requirements for OECTs varies depending on the application, with distinct requirements for brain signal recording compared to molecular sensing. Rapid operation ensures that the temporal dynamics of neural activity are preserved without distortion, enabling precise analysis and understanding of brain function and dysfunction. Also, high-frequency brain waves typically exhibit lower magnitudes compared to low-frequency oscillations.^29^ For the purposes of ECoG recordings, transistors must effectively amplify signals up to 500 Hz.^30^ The operational bandwidth of OECTs depends on several factors including channel width, length, thickness, and overlap of the channel with the source and drain.^31,32^

In addition, parasitic elements significantly contribute to the frequency limitation, notably influenced by the selection of the metal for transistor fabrication.^30^ Currently, inkjet microfabrication is constrained to utilizing gold or silver nanoparticles, with silver being predominant in the market.^33,34^ Our TLM data shown in Figure 3e demonstrates lower contact resistance for inkjet printed gold compared to silver. This difference is evident when analyzing the bandwidth of OECTs fabricated using both metals. For the two chosen thickness configurations −150 and 250 nm–gold OECTs presented with a wider bandwidth compared to silver OECTs. For instance, at 500 Hz, g_m_ dropped by 20% and 40% for gold and silver, respectively, in the 150 nm thickness configuration (Figure 3f). These results are attributed to the difference in the work function of the metals, gold and silver have work functions of 5.3 and 4.39 eV, respectively,^35^ while the Highest Occupied Molecular Orbital (HOMO) of PEDOT:PSS is approximately −5.5 eV.^36^ With gold demonstrating a closer alignment of work function and HOMO compared to silver, it is considered more favorable for developing PEDOT:PSS based OECTs. To strike a balance between g_m_ and speed we chose to proceed with the thicker 250 nm channel, in which the device experiences a decrease of 39% in gm from 1-to-500 Hz(Figure 3f, **inset**). The OECT’s transition from the On-state to the Off-state under a square wave is fitted to an exponentially decaying function with a time constant equal to the response time of the device and resulted in 0.5 ms (Figure 3g). Tests conducted using an Ag/AgCl probe yielded similar response times of 0.51 ms (Figure S5). The device’s On–Off current ratio, an indicator of SNR^30^ can also be extracted from the step response, On-current was chosen at −0.5 V_GS_ and was equal to −8.3 mA, Off-Current was chosen at 1.3 V_GS_ and was equal to −0.001 mA, resulting in an On–Off ratio of 8300. In Figure 3h and Table S1, we benchmark our inkjet OECT to mostly photolithography developed devices. With emphasis on the ones used in neural recordings, our device has the smallest channel area of 1950 μm^2^ (highest possible resolution for neural recording), is among the fastest with a response time of 0.5 ms, and has highest transconductance, reaching 11 mS all while being fabricated with a unique technique. Our results redefine the unreliable inkjet narrative^37^ by achieving consistent reproducibility through our process, optimizing the balance between size, speed, and amplification.

#### Response to Mechanical Stress

**Figure 4:**
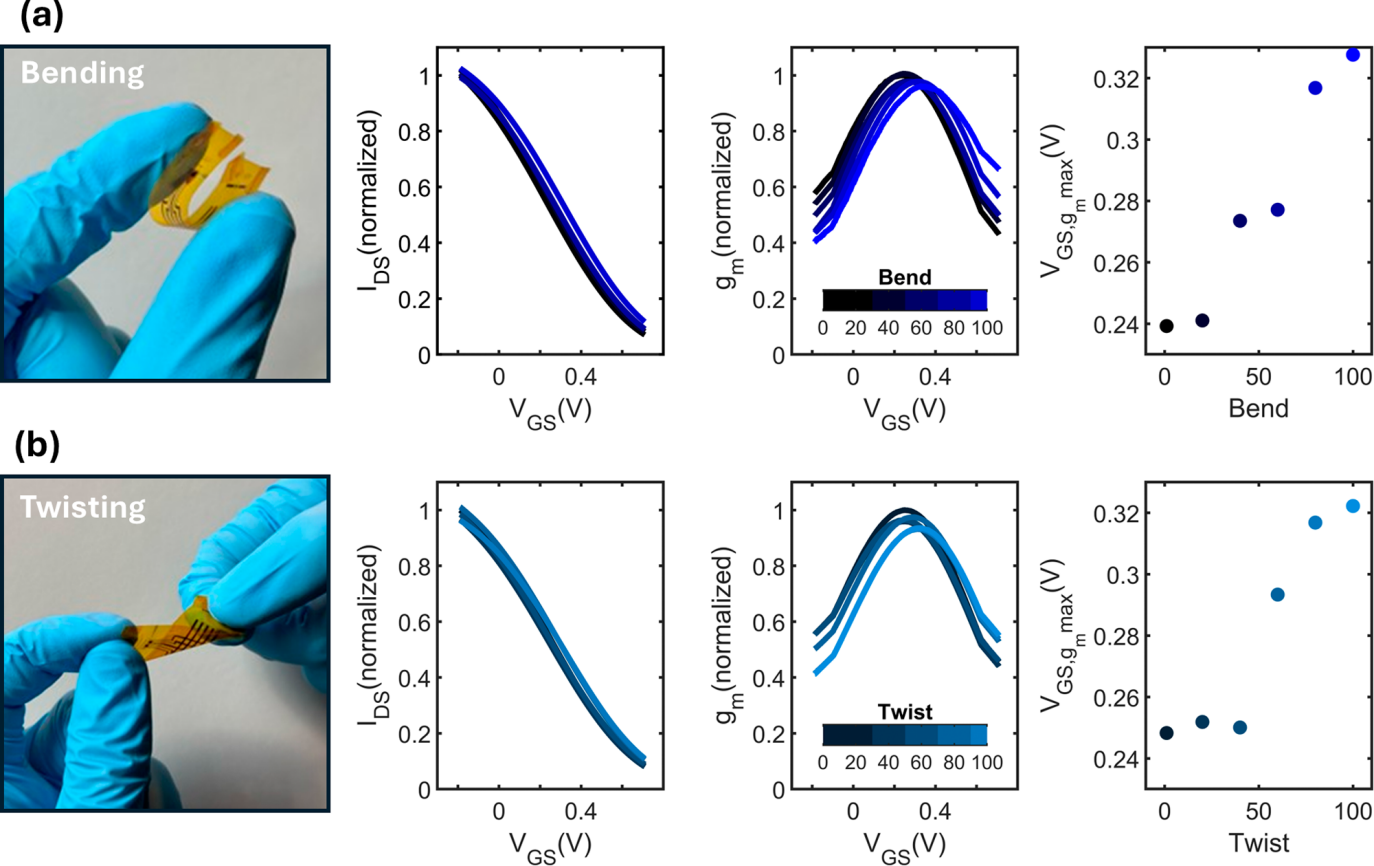
Electrical performance of OECTs under 100 (a) bending and (b) twisting cycles including transfer characteristics, g_m_ curves and location of peak g_m_.

To assess the impact of mechanical stresses on the performance of the printed device, a series of 100 bending (Figure 4a) and twisting (Figure 4b) cycles were applied to the developed films. Notably, g_m_ decreases slightly by 4% by the end of the bending cycles, and by 6% by the end of twisting cycles. In addition, V_GS,gm(max)_ steadily shifted to more positive values under both testing conditions. Our OECTs, made with gold, a highly malleable material, demonstrate significant resilience. Our tests showed that the devices retained their electrical performance even after several cycles, withstanding bending angles of over 150 degrees and full-range twisting (360 deg). Importantly, these testing conditions far exceed the stress and strains that the devices would encounter inside the brain, where bending and twisting would not occur at such extremes. These findings underscore the importance of considering mechanical stresses in the design and application of inkjet printed OECTs. While the observed decrease in g_m_ and shift in V_GS,gm(max)_ suggest some degradation in device performance over repeated bending and twisting cycles, the extent of these effects remains relatively modest. To preserve performance and mitigate detrimental effects, it is advisible to limit the usage of the devices to approximately 50 cycles of bending or twisting. The rightward shift in maximum amplification point is likely due to the formation of microcracks in the feeding lines. These microcracks increase impedance, consequently pushing the operating point to more positive gate voltage values.

#### Optimizing Gate Voltage

**Figure 5:**
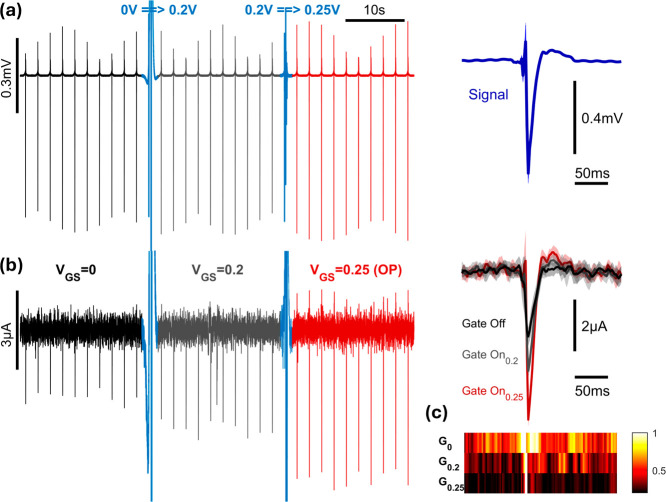
(a) Artificial neuronal spikes applied in solution (left) and the corresponding average (right). (b) Recorded signals using OECTs at different gate biases including 0 V_GS_ (labeled G_0_), 0.2 V_GS_ (labeled G_0.2_), and 0.25 V_GS_ (labeled G_0.25_) (left), and the corresponding average (right). (c) Color coded representation of the noise profile of the recorded signal at the different gate voltage settings.

The configuration of the OECT with a coated in-plane gate facilitates more effective modulation of the electric field within the channel region, optimizing the device for peak g_m_ operation. While in previous application of OECTs in neural recordings, authors did not include a planar gate to operate their OECTs at V_GS, gm max_,^14,38,39^ we demonstrate the effect of applying a bias in OECT neural interfaces through an in-plane integration. One key parameter to take into consideration when employing the gate bias is the accuracy of the gate DC voltage, ensuring it does not match that of the amplitude of the measured signal. In an initial benchtop experiment, the same voltage spike inputs simulating spiking neurons (Figure 5a), the behavior of the OECT changes drastically depending on the applied voltage at the gate. When the gate is shorted with the source, a maximum spike of −2.42 μA is recorded, note the inability to clearly characterize the upward deflection of the signal as it is masked by the noise. Partially turning on the gate, without placing the device in its optimal operating point, increases the amplitude of the signal by 55%, to record a maximum downward deflection of −3.75 μA, we still fail to correctly discern upward deflection in that setting as well, this simulates the incorrect use of the gate. However, at optimal operating voltage, we observe an increase in performance by 50% from the partial use of the gate, and 133% from 0 V_gs_, recording a maximum peak of −5.64 μA (Figure 5b). Additionally, because of the amplification factor introduced at that operating point, small upward deflections that might hold important biological information about the activity of the electroactive tissue can now be correctly represented by the output of the device, now having a stronger magnitude compared to the noise. Color coded representation of the normalized averaged signal standard deviation to the current of averaged peaks also reveals a quieter environment representing lower noise levels and higher SNR when operating the device at optimal gate voltage (Figure 5c). Furthermore, our findings underscore the significance of on-site amplification, selectively enhancing the signal of interest while minimizing noise interference. This stands in contrast to traditional amplifiers, which amplify the entire signal, including noise components.

### Low-Impedance Passive Electrodes for In Vivo Comparison

**Figure 6:**
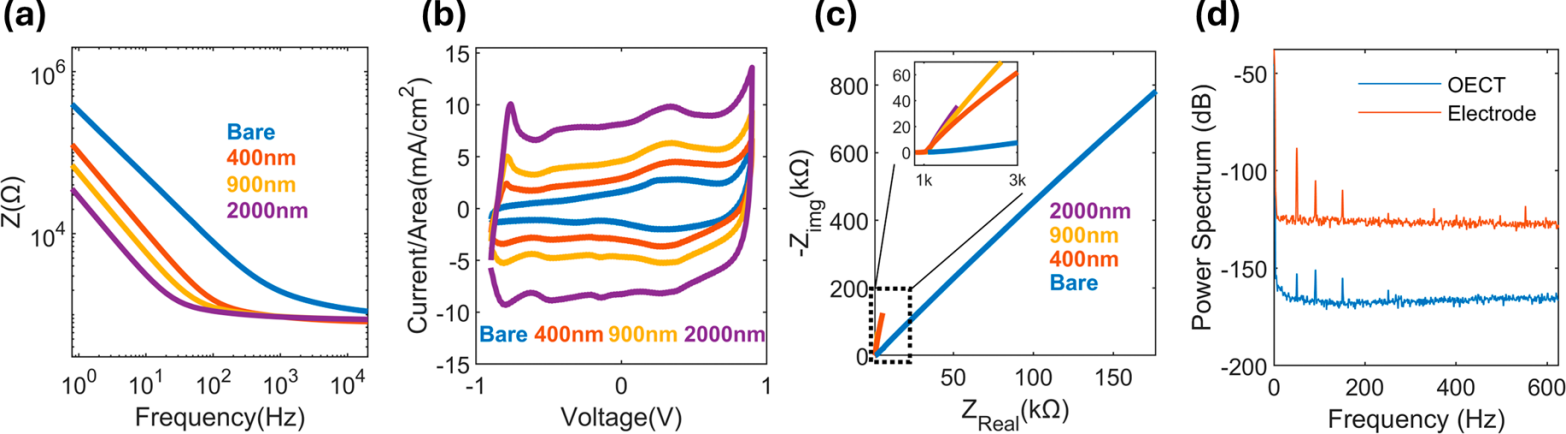
Electrochemical performance of bare gold and PEDOT:PSS coated (400, 900, and 2000 nm) gold Inkjet printed electrodes including (a) electrochemical impedance spectroscopy, (b) cyclic voltammetry and (c) Nyquist plot. (d) FFT spectrum of a 10 mV, 1 Hz sinusoidal arbitrary signal recorded simultaneously from an adjacent OECT (blue) and passive electrode (orange).

PEDOT:PSS-coated gold electrodes represent state of the art in electrocorticography (ECoG) electrode technology. The combination of gold’s conductivity with PEDOT:PSS’s electrochemical properties results in electrodes with superior performance characteristics. These electrodes exhibit reduced impedance, enhanced charge injection/extraction capacity, and improved long-term stability compared to conventional bare gold electrodes. All these parameters are further improved with inkjet printing given the thickness range of the process.^8^ In a secondary benchtop experiment we compare the performance of our Inkjet printed OECTS to ultralow impedance passive electrodes, 300 μm gold electrodes were patterned adjacent to the channel, and electrodes were modified by layering of PEDOT:PSS for improved performance. This layer-by-layer deposition method allows for fine control over the thickness and composition of the electrode coatings beyond microfabrication (thicknesses in the range of 50 to 300 nm versus inkjet printing allows for a wider range of thicknesses, typically from submicrometer to several micrometers), which can significantly enhance performance. The PEDOT:PSS coating decreased impedance significantly at low frequencies. At 100 Hz for example, the bare electrode and the 400 nm, 900 nm, 2000 nm coated electrodes had impedances of 7.8 kΩ, 1.5 kΩ, 1.2 kΩ, and 1.1 kΩ respectively (Figure 6a).

Bare gold electrodes exhibit minimal charge storage capacity, as evidenced by the reduced area under the cyclic voltammogram curve, whereas the PEDOT:PSS coating increased the current response of the system and expanded the area under the curves (Figure 6b), due to an increase in the charge storage capacity of the electrode.^40^

The corresponding Nyquist plots (Figure 6c) show typical behavior for the bare gold electrode,^41^ and the associated data for the coated variations also confirms the overall decrease in impedance, in addition to a prominent difference in two parameters, a decrease in the diffuse layer resistance, facilitating movement of ions at the electrode surface, and an increase in the slope of the curve, indicating the formation of a more prominent capacitance,^42^ expected by the addition of PEDOT:PSS (Figure 6c). To ensure overall uniformity, the OECT gate electrodes were coated with the same number of layers, totaling 900 nm in thickness. Sinusoidal signals with 10 mV_p-p_ and 1 Hz frequency applied at the gate were simultaneously recorded from both the OECT and electrode (Figure S6). AC recordings show that the OECT has a 20db higher SNR than the electrode (Figure 6d). Our comparison highlights the significant advantages of adopting a transistor over low-impedance PEDOT:PSS electrodes for recording electric activity inside an electrolyte.

### Active and Passive Devices Comparison in In Vivo Recordings of Seizures

Seizures are a type of abnormal brain activity characterized by sudden, excessive, and synchronous electrical discharges. ECoG recordings are crucial for diagnosing epilepsy, classifying seizures, and localizing seizure foci for potential surgical intervention, while also monitoring treatment efficacy and advancing research for seizure prediction and personalized therapy, ultimately improving outcomes and quality of life for patients with epilepsy. Such interventions however require the accurate localization of seizure foci with surface or intracerebral electrodes. Positioned on the surface of the brain, OECTs have been shown to provide information about the location and characteristics of seizures with performance matching that of more invasive penetrating electrodes.^13^

**Figure 7:**
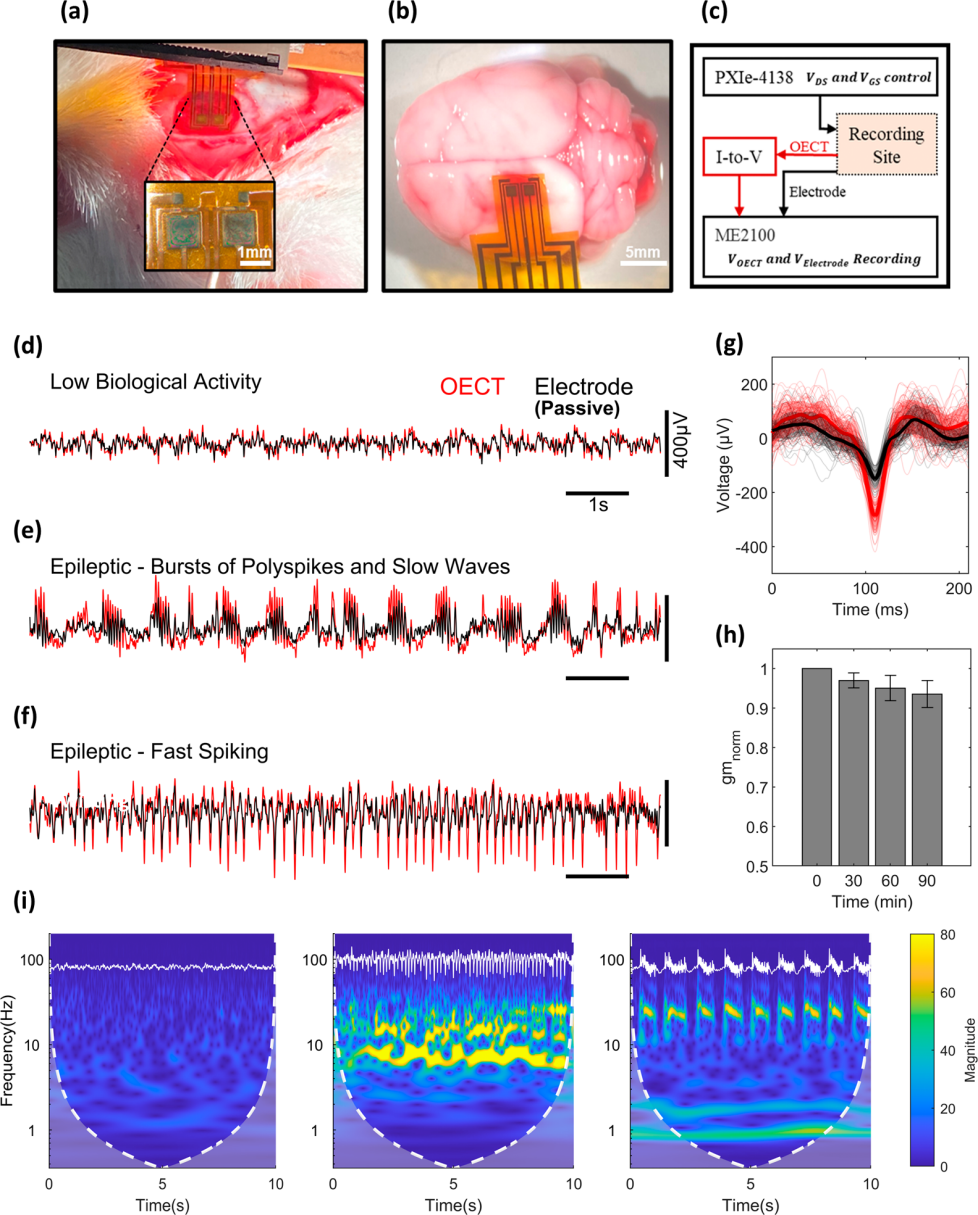
(a) Photographic illustration of a device implanted in vivo, having two OECTs and Electrodes. (b) Photographic illustration of the site of recording. (c) Block diagram of the in vivo recording setup. (d) Overlaid OECT and electrode voltage signals during low biological activity, (e) Epileptic bursts and (f) spikes episodes. (g) Averaged recorded spikes of adjacent OECT and electrode during an epileptic spike episode. (h) Normalized gm of four devices during a period of 90 min to assess stability of the channel inside an electrolyte. (i) Scalograms of recorded OECT signals during the distinct brain activities, low activity (left), fast spiking (middle), and spiking bursts (right).

To develop our implantable device, OECTs were Inkjet-printed on polyimide sheets, with an active recording site of 3.8 × 1.6 mm, containing two OECT units in a common source configuration and two passive electrodes (Figure 7a). Connection pads, the size of 0.6 × 3 mm, were also printed to connect the device to the acquisition system through a Zero Insertion Force (ZIF) board. The ZIF board was secured to a stereotaxic arm and positioned right above the recording site. Lowering the arm allowed the device to rest on the outer cortex of the left hemisphere, centered on the sensory-motor cortex (Figure 7b). The experiment required two systems working in parallel, one to control the voltage supply to the transistors through the use of a total of four source measuring units (SMU) PXIe-4138, two for gate control and two for drain-source voltage control. Also, an ME2100 acquisition unit was used to collect the signals generated by the OECTs and electrodes. To unify the unit of the recorded signals, a 220-ohm resistor was connected in series with the drain of both OECTs to transform readings from current to voltage (Figure 7c). To conduct the in vivo recordings, seizures were induced in rats through the administration of (KA), a neurotoxic analog of glutamate.^43^ Injection of KA in the basolateral amygdala gave rise to electrographic seizures occurring after 20 ± 5 min. Signals were recorded by the OECT and the adjacent passive electrodes and divided into three categories, which were previously described in KA-induced limbic seizures^20,44^: **(1)** baseline slow biological activity in which signals are relatively low in magnitude and slow (<3–4 Hz) (Figure 7d), **(2)** then, the seizures started with bursts of polyspikes and slow wave discharges identified by tightly packed 25 ± 5 Hz polyspikes lasting approximately 0.8 s and occurring around once every second (Figure 7e), **(3)** the seizure then evolved into the second seizure pattern consisting of fast spiking characterized by pointed waves at a frequency of 10 Hz (Figure 7f). In all three signal types, OECTs show clear superiority in recording brain activity in terms of signal amplitude, achieving a peak amplification of around 76% relative to the electrode (Figure 7g). In addition to amplifying the signal, SNR was also improved. It was calculated following eq 5 below:
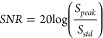
5Where *S*_*peak*_ is the highest peak during an epileptic episode and *S*_*std*_ is the standard deviation of a period of low biological activity. SNR scored 48 dB for OECTs and 38 dB for passive electrodes in recording spikes, and 42.7 dB for OECTs against 35 dB for electrodes in recording bursts. Concerns are typically made when using PEDOT:PSS films in solution as they are not very stable in water mainly due to swelling, delamination, and decrease of conductivity with time, limiting the use of the device to acute recordings. To demonstrate the stability of the inkjet printed channel, g_m_ was extracted from four devices immersed in solution for a duration exceedissng that of the acute experiments. On average, gm dropped by 6.5% by the end of the test (Figure 7h). Wavelet transforms of the normal biological activity and the two seizure patterns detected are depicted in Figure 7i.

## Conclusions

The development of high-fidelity biopotential recording devices is crucial for accurately capturing physiological signals, which enables precise clinical diagnoses and supports real-time applications such as brain-machine interfaces. Our work demonstrates the significant advancements in the field of organic electrochemical transistors (OECTs) for electrophysiological monitoring. By employing a novel three-step drop-on-demand (DoD) inkjet printing process, we successfully fabricated fully planar, high-performance OECTs on flexible substrates with exceptional reproducibility. This technique significantly streamlines the fabrication process, reducing both the cost and environmental footprint compared to traditional methods. The resulting device not only features high transconductance and rapid response times but also maintains a compact channel area, enhancing the spatial resolution required for precise neural recordings.

The developed PEDOT:PSS OECT devices exhibits a high gm and a rapid response time while maintaining a compact channel area for high spatial resolution neural recordings. In addition, the device is optimized with a planar PEDOT:PSS coated gold gate, and when correctly operated ensures at peak gm operation, resulting in optimal performance.

Our findings indicate that the inkjet-printed OECTs are particularly effective in recording distinct phases of seizures in a rat model, demonstrating superior performance with a high signal-to-noise ratio (SNR) of up to 48 dB compared to low-impedance passive electrodes. This demonstrates the potential of OECTs to revolutionize neurological diagnostics and lays the groundwork for further exploration of their application in other medical diagnostics and biopotential sensing technologies. These results underscore the reliability and effectiveness of inkjet-printed OECTs, especially in applications requiring high spatial resolution, rapid response, and precise signal amplification, thereby challenging the narrative of inkjet printing's limitations. Moving forward, the implications of this work suggest a promising future for OECTs in the realm of biomedical sensing and diagnostics.

## Supporting Information Available

Theory of operation, inkjet printing waveforms, morphological analysis of inkjet printed gold, transfer characteristics forward and backward scan, OECT response time using an Ag/AgCl external gate electrode, comparison of our device to existing OECTs in literature, simultaneous recording from electrode and OECT under arbitrary signal (PDF)

## ABBREVIATIONS

OECT: organic electrochemical transistor
DS: drop spacing
SNR: signal to noise ratio
SMU: source measure unit
ZIF: zero insertion force
PEDOT:PSS: poly(3,4-ethylenedioxythiophene)-poly(styrenesulfonate)
IACUC: Animal Care and Use Committee
