# Supplementary material for: Inkjet-Printed, Flexible Organic Electrochemical Transistors for High-Performance Electrocorticography Recordings
Source: ACS Appl Mater Interfaces. 2024 Aug 15;16(41):55045–55. doi: 10.1021/acsami.4c07359 (PMC11492168; doi:10.1021/acsami.4c07359)
Supplement: Supplementary file 1 — am4c07359_si_001.pdf [file am4c07359_si_001.pdf]

Supporting Information

Inkjet-Printed, Flexible Organic Electrochemical Transistors for High-Performance Electrocorticography Recordings

Fadi Khoury [1], Sahera Saleh [1], Heba Badawe [1], Makram Obeid [2], Massoud Khraiche [1,*]

[1] Neural Engineering and NanoBiosensors Group, Biomedical Engineering Program, Maroun Semaan Faculty of Engineering and Architecture, American University of Beirut, Beirut 1107 2020, Lebanon.
[2] Stark Neurosciences Research Institute, Department of Neurology, Indiana University School of Medicine, Indianapolis, IN 46202, United States of America.

*Corresponding author at:

Dr. Massoud Khraiche: Neural Engineering and NanoBiosensors Group, Biomedical Engineering Program, Maroun Semaan Faculty of Engineering and Architecture, American University of Beirut, Beirut 1107 2020, Lebanon

E-mail: mkhraiche@aub.edu.lb

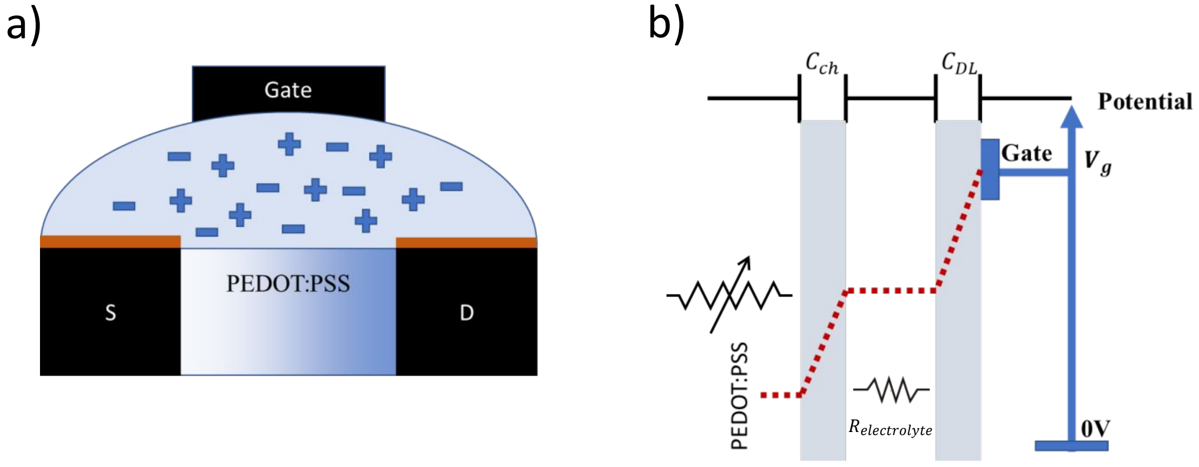

Figure S1 *Theory of operation. (a) schematic of the ionic environment in which the organic electrochemical transistor operates. (b) equivalent circuit and voltage drop representation*

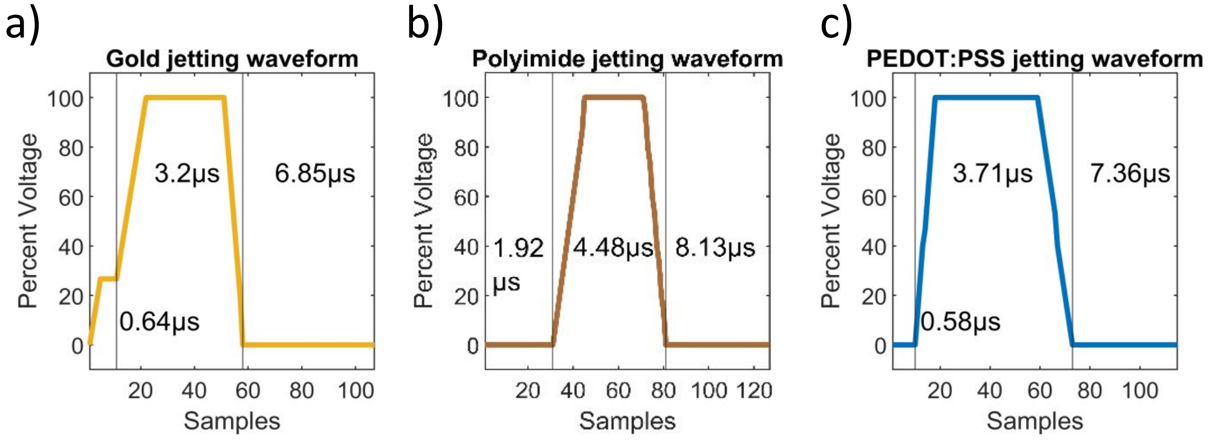

Figure S2 *Inkjet printing waveforms used for the deposition of (a) gold, (b) insulating polyimide, (c) semiconducting PEDOT:PSS*

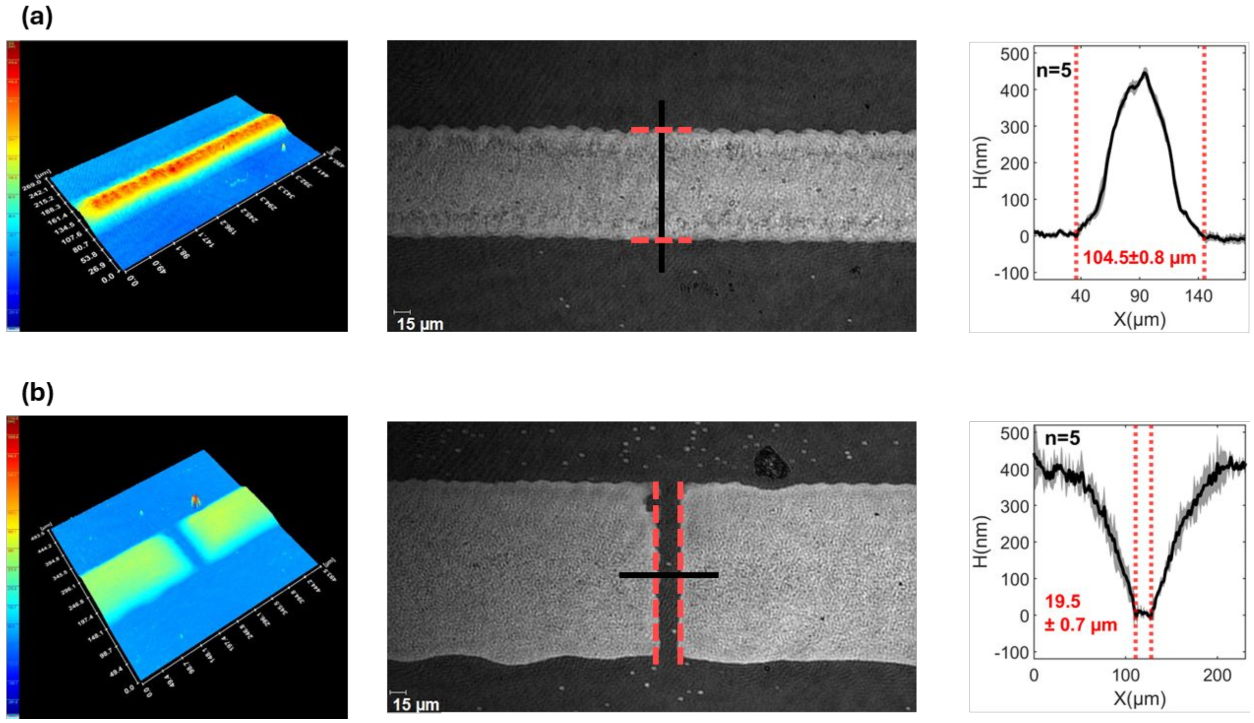

Figure S3 *morphological analysis of inkjet accuracy using DHM. 3D holographic reconstruction (left), phase image with profiling lines highlighted in black and delimiters in red (middle), and corresponding profile (right) for (a) width and (b) 1 pixel separation of gold leads.*

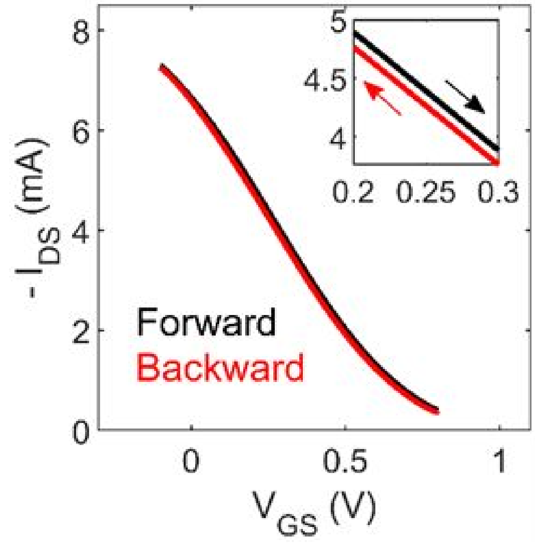

Figure S4 *forward and backward scans of transfer characteristics.*

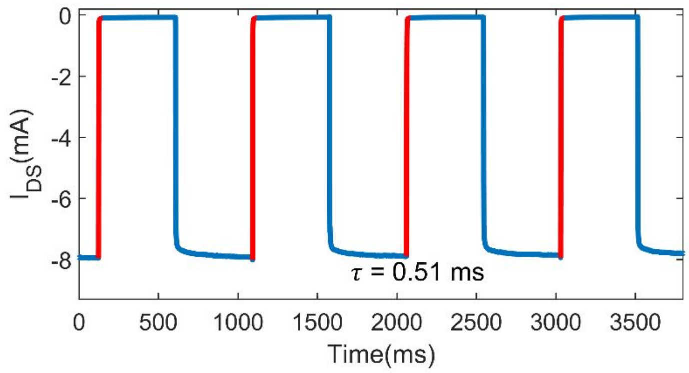

Figure S5 *OECT response time using an Ag/AgCl external gate electrode*

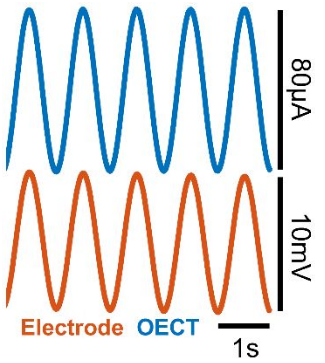

Figure S6 *simultaneous recording from the fabricated low impedance electrode (blue), and OECT (orange) under a 10 m*$V_{p\text{-}p}$ *,1Hz sinusoidal signal.*

***Table S1** comparison of our developed device to existing high performing OECTs in literature.*

Study	gm (ms)	Response time (ms)	Width W(um)	Length L(um)	Thickness d(um)	Wd/L	Channel Area (um^2)	Fabrication Technique	Application
Our Work	11	0.5	130	15	0.25	2.16	1950	Inkjet Printing	Brain
[1]	1.1	0.363	90	60	0.15	0.225	5400	Photolithography	Brain
[2]	0.9	N/A	15	6	N/A	N/A	90	Photolithography	Brain
[3]	9	1.42	200	20	N/A	0	4000	Photolithography	Brain
[4]	5	0.32	50	50	500	500	2500	Photolithography	Brain
[5]	2.5	0.15	40	30	N/A	0	1200	Photolithography	Cardiac
[6]	6	0.102	38	24	0.115	0.182	912	Photolithography	Cardiac
[7]	1.1	0.06	10	10	N/A	0	100	Photolithography	Cardiac
[8]	10	0.1	78	86	0.035	0.031	6708	Photolithography	In vitro
[9]	2	0.062	25	25	0.1	0.1	625	Photolithography	Intracellular AP
[10]	28.7	N/A	1000	200	0.8	2	200000	Lithography	N/A
[10]	14.2	3.5	1000	100	0.2	2	100000	Lithography	N/A
[10]	30.5	9.9	1000	100	0.4	4	100000	Lithography	N/A
[11]	4	0.09	100	10	0.2	2	1000	Photolithography	N/A, not PEDOT:PSS
[12]	12.9	0.179	1000	100	0.101	1.01	100000	Spray Deposition	N/A
[13]	2	N/A	10	5	0.14	0.28	50	Photolithography	N/A

References

(1) Lee, W.; Kim, D.; Matsuhisa, N.; Nagase, M.; Sekino, M.; Malliaras, G. G.; Yokota, T.; Someya, T. Transparent, conformable, active multielectrode array using organic electrochemical transistors. *Proceedings of the National Academy of Sciences of the United States of America* **2017**, *114* (40), 10554-10559. DOI: 10.1073/pnas.1703886114.
(2) Khodagholy, D.; Doublet, T.; Quilichini, P.; Gurfinkel, M.; Leleux, P.; Ghestem, A.; Ismailova, E.; Hervé, T.; Sanaur, S.; Bernard, C.; et al. In vivo recordings of brain activity using organic transistors. *Nature Communications* **2013**, *4*. DOI: 10.1038/ncomms2573.
(3) Wu, M.; Yao, K.; Huang, N.; Li, H.; Zhou, J.; Shi, R.; Li, J.; Huang, X.; Li, J.; Jia, H.; et al. Ultrathin, Soft, Bioresorbable Organic Electrochemical Transistors for Transient Spatiotemporal Mapping of Brain Activity. *Advanced Science* **2023**, *10* (14). DOI: 10.1002/advs.202300504.
(4) Rivnay, J.; Leleux, P.; Ferro, M.; Sessolo, M.; Williamson, A.; Koutsouras, D. A.; Khodagholy, D.; Ramuz, M.; Strakosas, X.; Owens, R. M.; et al. High-performance transistors for bioelectronics through tuning of channel thickness. *Science Advances* **2015**, *1* (4). DOI: 10.1126/sciadv.1400251.
(5) Gu, X.; Yao, C.; Liu, Y.; Hsing, I. M. 16-Channel Organic Electrochemical Transistor Array for In Vitro Conduction Mapping of Cardiac Action Potential. *Advanced healthcare materials* **2016**, *5* (18), 2345-2351.
(6) Liang, Y.; Ernst, M.; Brings, F.; Kireev, D.; Maybeck, V.; Offenhäusser, A.; Mayer, D. High Performance Flexible Organic Electrochemical Transistors for Monitoring Cardiac Action Potential. *Advanced Healthcare Materials* **2018**, *7* (19). DOI: 10.1002/adhm.201800304.
(7) Lee, W.; Kobayashi, S.; Nagase, M.; Jimbo, Y.; Saito, I.; Inoue, Y.; Yambe, T.; Sekino, M.; Malliaras, G. G.; Yokota, T. Nonthrombogenic, stretchable, active multielectrode array for electroanatomical mapping. *Science advances* **2018**, *4* (10), eaau2426-eaau2426.
(8) Tyrrell, J. E.; Boutelle, M. G.; Campbell, A. J. Measurement of Electrophysiological Signals In Vitro Using High-Performance Organic Electrochemical Transistors. *Advanced Functional Materials* **2021**, *31* (1). DOI: 10.1002/adfm.202007086.
(9) Jimbo, Y.; Sasaki, D.; Ohya, T.; Lee, S.; Lee, W.; Arab Hassani, F.; Yokota, T.; Matsuura, K.; Umezu, S.; Shimizu, T. An organic transistor matrix for multipoint intracellular action potential recording. *Proceedings of the National Academy of Sciences* **2021**, *118* (39), e2022300118-e2022300118.
(10) Wu, X.; Surendran, A.; Ko, J.; Filonik, O.; Herzig, E. M.; Müller-Buschbaum, P.; Leong, W. L. Ionic-liquid doping enables high transconductance, fast response time, and high ion sensitivity in organic electrochemical transistors. *Advanced Materials* **2019**, *31* (2), 1805544-1805544.
(11) Inal, S.; Rivnay, J.; Hofmann, A. I.; Uguz, I.; Mumtaz, M.; Katsigiannopoulos, D.; Brochon, C.; Cloutet, E.; Hadziioannou, G.; Malliaras, G. G. Organic electrochemical transistors based on PEDOT with different anionic polyelectrolyte dopants. *Journal of Polymer Science Part B: Polymer Physics* **2016**, *54* (2), 147-151.
(12) Wu, X.; Surendran, A.; Moser, M.; Chen, S.; Muhammad, B. T.; Maria, I. P.; McCulloch, I.; Leong, W. L. Universal spray-deposition process for scalable, high-performance, and stable organic electrochemical transistors. *ACS applied materials & interfaces* **2020**, *12* (18), 20757-20764.
(13) Rivnay, J.; Leleux, P.; Sessolo, M.; Khodagholy, D.; Hervé, T.; Fiocchi, M.; Malliaras, G. G. Organic electrochemical transistors with maximum transconductance at zero gate bias. *Advanced Materials* **2013**, *25* (48), 7010-7014. DOI: 10.1002/adma.201303080.
